# Ocular Symptomatology, Management, and Clinical Outcome of a Giant Intracranial Aneurysm

**DOI:** 10.1155/2012/643965

**Published:** 2012-04-03

**Authors:** Chryssa Terzidou, Georgios Dalianis, Fani Zacharaki

**Affiliations:** ^1^Department of Ophthalmology, Patission General Hospital, 15-17 Halkidos Street, 11143 Athens, Greece; ^2^Department of Ophthalmology, University Hospital of Larissa, Mezourlo, 41110, Larissa, Greece

## Abstract

Giant aneurysms of the anterior intracranial circulation are rare, slowly progressive vascular abnormalities, often presenting with neuro-ophthalmological symptoms before they rupture. This is a case of a 55-year-old woman with a double aneurysm of the anterior intracranial circulation, part of which was giant, diagnosed exclusively on the basis of ocular manifestations. We also describe successful management of the case throughout a long follow-up period.

## 1. Introduction

Intracranial aneurysms, most of which asymptomatic, are estimated to be present in approximately 5% of the population [1, 2]. The most serious complication of intracranial aneurysms is subarachnoid hemorrhage, which results in death in 50% of cases. Larger aneurysms are considered to carry a higher rupture risk, especially when located in the posterior circulation [3]. Small (<10 mm) and medium (10–25 mm) aneurysms tend to rupture without previous symptomatology, whereas giant aneurysms (diameter >25 mm) have a different natural course, progress slowly, and usually (in 50% of patients) produce neurological symptoms, depending on the location, size, and the way of expansion [4, 5].

## 2. Case Presentation

**Figure 1:**
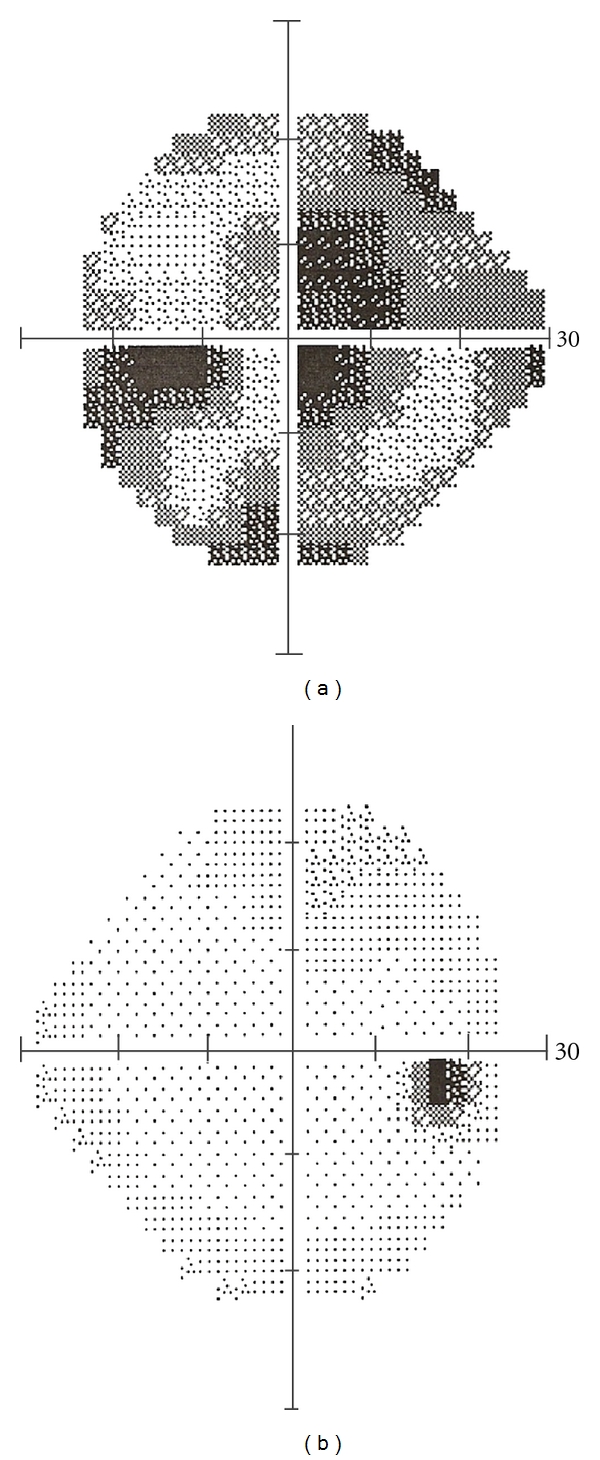
Visual fields of the patient at presentation. The RE is normal, and the LE shows a deep central scotoma (V/A 20/40).

A 55-year-old female presented complaining of reduced visual acuity in the left eye that had been progressing for 5 months. She had no remarkable medical history and was not receiving any medical treatment. Visual acuity in the Snellen chart was 20/20 in the right eye and 20/40 in the left eye. Pupillary responses were normal in both eyes whereas color vision, tested with the Ishihara charts, was significantly disturbed in the left eye. Slit lamp examination and intraocular pressure were normal in both eyes. Dilated fundus examination disclosed no pathology. Visual fields examination (Humphrey 24-2 SITA standard) was normal in the right eye and revealed a deep central scotoma in the left eye (Figure 1). The patient refused to undergo the suggested MRI examination, at that time.

 Twenty days later, the patient returned with further loss of vision in her left eye. Ocular examination revealed visual acuity in the left eye 20/80 and a relatively afferent pupillary defect (RAPD). Fundus examination disclosed partial atrophy of the left optic nerve.

**Figure 2:**
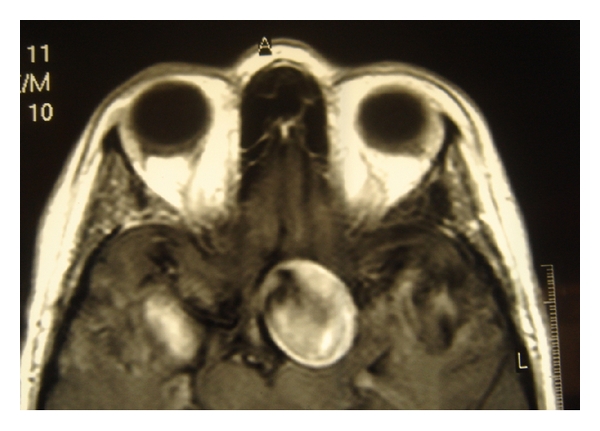
Axial T1-weighted MRI revealing the giant internal carotid aneurysm.

**Figure 3:**
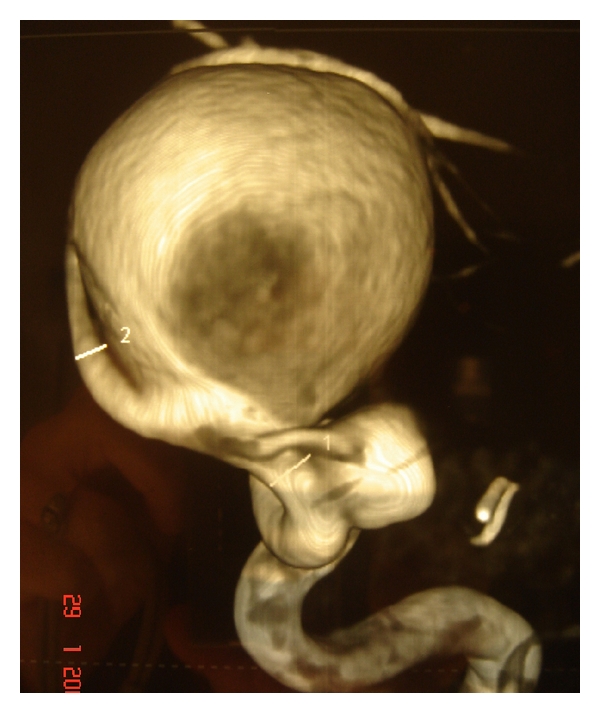
3-dimensional digital angiography illustrating the double aneurysm.

**Figure 4:**
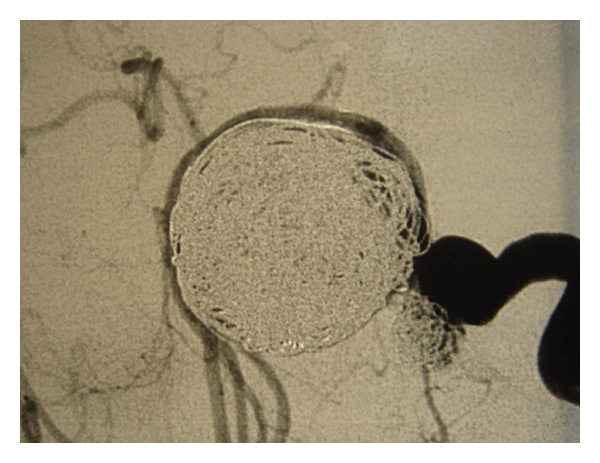
Digital angiography of the aneurysms after the second successful embolization.

 Emergency MRI and consequent selective digital angiography confirmed the presence of a giant aneurysm of 28 mm of diameter, arising from the supraclinoid part of the left internal carotid and a second smaller aneurysm of 9 mm diameter with a wide base near the origin of the left ophthalmic artery (Figures 2 and 3). The patient was referred to the Department of Neurosurgery where successful endovascular embolism of the aneurysms was performed, leaving the left internal carotid blood flow intact. Three months later, the patient presented with worsening of vision in her left eye, which was assessed to 20/400. The new MRA showed late failure of the previous embolization procedure, due to recanalization of the aneurysm. An additional, totally successful, embolization was performed (Figure 4).

**Figure 5:**
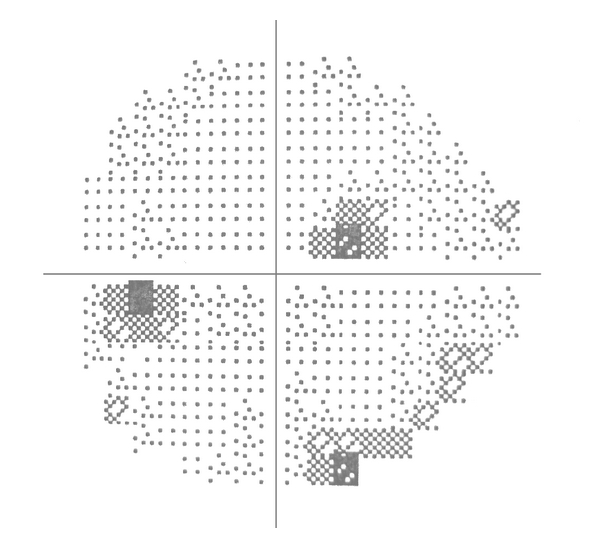
Visual field of the LE 20 months after successful embolism (V/A 20/25).

The patient's symptoms gradually recessed, and, at 20 months followup, visual acuity is 20/25 with significant improvement of visual fields findings (Figure 5). Partial atrophy of the left optic nerve is a constant finding throughout follow-up period.

## 3. Discussion

Giant aneurysms are most frequently located in the anterior part of the cerebral circulation (75%) and may induce severe pressure on one or both optic nerves, resulting in visual field and visual acuity disturbances [5, 6]. Diplopia due to third nerve palsy may occur owing to either expansion of the aneurysmal sac or rupture of the aneurysm. Intraocular hemorrhage is a rare complication.

 Supraclinoid aneurysms, arising from the internal carotid artery distal to the ophthalmic artery and proximal to the posterior communicative artery junction, tend to present late, usually with progressive visual loss rather than rupture [7]. Like in our case, patients typically have visual complains and visual field defects. Monocular visual field defects are most common [8].

 Management of giant aneurysms is controversial. Treatment options include microsurgical clipping, endovascular treatment, and combined techniques and observation. Conservative treatment leads to a 60% mortality rate within 2 years from diagnosis [9].

 Surgical treatment offers patients immediate relief of pressure, by deflating the aneurysmal sac, which is crucial in order to avoid permanent loss of visual ability in certain patients. On the other hand, microsurgical treatment is related to higher rates of postoperative morbidity, depending on patient's age and general condition [3, 9]. A recently published series of surgically treated giant aneurysms reports 13% surgical mortality and 9% permanent neurological morbidity, with overall good outcome in 81% of cases [10]. Therefore, treatment is usually individualized [9, 11].

 Coiling relieves pressure more gradually, by replacing the volume of the aneurysm. In a series of 321 unruptured aneurysms that underwent endovascular embolization, overall mortality was estimated to be 1.7%. Total occlusion of the aneurysm was accomplished in 68.5% of patients and subtotal in 27.8% [12]. Technical failure appeared to be the most significant complication of endovascular treatment, leading to secondary treatment in 3.2% of cases. A recent meta-analysis of endovascular treatment of intracranial unruptured aneurysms estimated that retreatment is necessary in 9.1% of cases [13].

 In our case, visual function, as expressed by visual acuity and visual fields measurements, showed dramatic improvement following successful retreatment. This favorable outcome is consistent with the literature showing the importance of prompt treatment. Best results, with improvement of vision, are obtained when patients are operated within a few months from the first symptoms [5].

Giant cerebral aneurysms, though rare, can be a cause of mortality and morbidity. Since they give no intense early symptomatology, high clinical suspicion is required in cases of inexplicable reduction in visual acuity or atypical symptoms. Prompt recognition and treatment is mandatory to preserve patient's life and visual function.
